# Agreement of Self-Reported and Administrative Data on Employment Histories in a German Cohort Study: A Sequence Analysis

**DOI:** 10.1007/s10680-018-9476-2

**Published:** 2018-03-21

**Authors:** Morten Wahrendorf, Anja Marr, Manfred Antoni, Beate Pesch, Karl-Heinz Jöckel, Thorsten Lunau, Susanne Moebus, Marina Arendt, Thomas Brüning, Thomas Behrens, Nico Dragano

**Affiliations:** 1 Centre for Health and Society, Institute for Medical Sociology, Faculty of Medicine, University of Düsseldorf, Universitätsstrasse 1, 40225 Düsseldorf, Germany; 2 Institute for Medical Informatics, Biometry, and Epidemiology, Faculty of Medicine, University Duisburg-Essen, Essen, Germany; 3 Institute for Employment Research (IAB), Nuremberg, Germany; 4 Institute for Prevention and Occupational Medicine of the German Social Accident Insurance (IPA), Institute of the Ruhr University Bochum, Bochum, Germany

**Keywords:** Comparison, Survey data, Administrative data, Employment histories, Sequence analysis

## Abstract

Collecting life course data is increasingly common in social and epidemiological research, either through record linkage of administrative data or by collecting retrospective interview data. This paper uses data on employment histories collected through both strategies, compares the attained samples, and investigates levels of agreements of individual histories. We use data from the German Heinz Nixdorf Recall Study with information on employment histories collected retrospectively from 2011 until 2014 (*N* = 3059). Administrative data from the German Institute for Employment Research (IAB) were linked to the survey data. After comparing respondents who provide self-reported histories with the subsample of the ones for which administrative data were available, we investigate the agreement of individual employment histories from the two sources (between 1975 and 2010) using sequence analyses. Almost all participants provided survey data on employment histories (97% of the sample), linkage consent was given by 93%, and administrative data were available for 63% of the participants. People with survey data were more likely to be female, to have a higher education, and to work self-employed and in the tertiary sector. The agreement of individual employment histories is high and similar across time, with a median level of agreement of 89%. Slightly lower values exist for women and people working in the tertiary sector, both having more complex histories. No differences exist for health-related factors. In conclusion, it is likely that missing consent and failed record linkage lead to sample differences; yet, both strategies provide comparable and reliable life course data.

**Electronic supplementary material:**

The online version of this article (10.1007/s10680-018-9476-2) contains supplementary material, which is available to authorized users.

## Introduction

There is increasing interest in understanding the impact of life course conditions on later outcomes, for example of work and employment on health in older ages (Kuh et al. 2003; Dannefer 2003; Vanhoutte and Nazroo 2015; Blane et al. 2016). The interest, hereby, is not only to know whether a person once worked in a job under specific conditions during their working life, but also to collect data on complete employment histories. Yet, data to answer these questions are rare and require detailed information. An attempt to overcome this limitation in existing studies is to collect information retrospectively, with generally two approaches: first, by linking administrative data on employment histories to existing survey data (data linkage), or second, by asking respondents to recall their employment histories. Extensive literature describes the advantages and disadvantages of both strategies (Belli et al. 2007; Manzoni et al. 2010; Korbmacher and Schroeder 2013; Herzog et al. 2007; Giele and Elder 1998; Antoni and Seth 2012). Survey data rely on the ability and willingness to recall previous employment histories, with a tendency to simplify and reduce complexity (Rubin and Baddeley 1989; Sudman et al. 1996; Solga 2001). Administrative data, in turn, usually require respondent’s consent (consent that is usually not given by all respondents), and additionally, the data are not always available for each respondent of the study, such that linkage is possible for part of the sample only (Jenkins et al. 2006; Korbmacher and Schroeder 2013; Sakshaug and Antoni 2017). Yet, while previous studies have used either of these two approaches, hardly any studies provide both types of data. As such, direct comparisons of recalled data with administrative records are still limited. These comparisons help to illustrate the differences, advantages and disadvantages of both strategies. Such knowledge is instrumental because it is relevant for studies aiming at collecting life history data.

Using data from a German cohort study (Heinz Nixdorf Recall Study), with data on recalled and administrative employment histories (in terms of annual information on employment circumstances between 1975 and 2010 (36 time points)), we have three objectives: First, we contrast respondents who provide survey data on recalled histories and those for whom administrative data are available, in terms of socio-demographic, health and work-related characteristics. Thus, we ask if collecting data via record linkage or via survey data leads to selective samples. Because of required consent, and because employment information is only available for episodes as a salaried employee in the administrative data we expect a smaller sample, dominated by people who worked as salaried employees in the case of administrative data. As a second aim, we compare entire histories for those who provide information from both sources and investigate how similar these histories are. In addition to conventional methods (e.g., testing overlaps for each year between 1975 and 2010 separately), this is done on the basis of sequences analysis (Studer and Ritschard 2016; Aisenbrey and Fasang 2010). In doing so, we do not investigate if singular reported jobs match between the two sources, but rather contrast entire employment histories from the two sources and, as a third aim, investigate if levels of agreement depend on socio-demographic, health and work-related variables (including job sectors). Here, we may assume that agreements are generally higher for men or job sectors for which employment histories are less complex. Our study adds to the literature by providing evidence on possible sample selectivity, and more importantly, by investigating agreements of recalled and administrative employment histories.

## Methods

### Sample

We use data from the Heinz Nixdorf Recall (Risk Factors, Evaluation of Coronary Calcium and Lifestyle) study (HNR study), a prospective cohort study conducted in three cities of the German Ruhr area (Schmermund et al. 2002). The HNR study was originally designed to investigate and evaluate established and new predictors of coronary heart diseases, including social and occupational risk factors. The baseline sample of men and women aged 45 to 75 years is drawn via probability simple random selection (stratified by cities) based upon mandatory local registries. Data are collected at the Examination Centre located at the University Clinic in Essen, using self-administrated questionnaires, computer-assisted personal interviews (CAPI) and clinical examinations. The collection meets high-quality standards, including trained interviewers and standardized procedures. Baseline data collection was 2000–2003, with two subsequent waves (follow-ups) in 2006–08 (wave 2) and 2011–2014 (wave 3). At the onset of the study, the response rate was 56%, with a total sample size of 4818 respondents (Stang et al. 2005). The attrition rate between baseline and the second wave is 10%, and data from wave 3 are available for 3059 respondents. In contrast to wave one and two, the interview of wave 3 also includes a retrospective questionnaire collecting data on previous employment histories. The interview also collects all necessary information and consent for linkage of administrative data. This serves the aim to collect life history data, and to develop and explore different strategies of gaining retrospective information in the frame of epidemiological surveys. Approval for the study was obtained from the ethical commission of the Medical Faculty at the University of Duisburg-Essen. More details of the study can be found in other literature (Schmermund et al. 2002).

### Self-Reported Employment Histories

The third wave collects information on previous employment histories based on CAPI. In preparation for the interview, respondents were asked to make brief notes on each job of their working lives (lasting 6 month or longer). This served as reminder to collect the following details in the interview: the year when a job started and ended, working hours, job sector, working contract (permanent vs. fixed-term employment), the employment status (self-employed or salaried employment), and an open question about the job title with a brief description of the job task (as a basis to recode and classify jobs). As a result, we can derive individual employment sequences with annual information on the individual employment situation for each year of age between the age they were when they started their first job and the time of data collection (often more than 50 years). Among those who were interviewed in wave 3 (3059 respondents) 97% provided self-reported data on employment histories (2983 respondents).

### Administrative Employment Histories

The administrative data of the Institute for Employment Research (“IAB”) are based on employment records from the German Federal Employment Agency (“Bundesagentur für Arbeit”) (Antoni et al. 2016). Each person who has at least one job episode as salaried employee (involving social security contributions) is part of these records. Records rely on the mandatory German notification scheme (so-called DEÜV-notification procedure as established in the year 1973), obliging each employer since 1975 to give information on their employees at least once a year (as a basis to calculate pension and unemployment entitlements). The yearly recorded information contains, among others, the precise date when a job started and ended, a title of the occupation (based on a national classification scheme), information on working hours (part-time vs. full-time job), and whether the work is part of vocational training. People who never worked as a salaried employee, or those who were always self-employed or worked as a civil servant, do not appear in the records. Also, even if someone appears in the data, but once worked self-employed or as a civil servant, this latter job episode is not recorded.

To enable record linkage, the HNR study applied strict rules of data protection: Respondents were first informed about the planned linkage and asked for written consent in the interview as well as additional information necessary for record linkage (Social Security number (SSN), name, date of birth and last employer). The forms with linkage identifiers were stored securely at the study center and delivered to the IAB on a regular basis, where staff members entered this information into a database. Then IAB staff derived administrative data in an iterative procedure: If a valid SSN was available, the respondent’s administrative record could be drawn directly. In the case that respondents did not provide a valid SSN, the linkage was based on alternative procedures, for example, according to birth date or name or both (see Sakshaug et al. (2017) for an exemplary record linkage application with IAB data). Thereafter, the extracted and de-identified data on employment histories were delivered back to the study center in Essen, where it was merged with the self-reported data. Procedures were approved by the review boards of the German Ministry of Labour and Social Affairs. Data are available from 1975 to 2010 (36 years), and we can again derive individual employment sequences. Among those who were interviewed in wave 3 (3059 respondents), administrative data are available for 63% (1927 respondents). This is due to missing consent (valid consent was available for 2836 respondents (93%) out of 3059 respondents) and also due to failed record linkage (linkage was possible for 2202 (78%) out of 2836 respondents with consent). This is below other linkage rates of the IAB (Sakshaug et al. 2017) and may have different reasons: In addition to insufficient information for linkage (e.g., invalid SSN), it is also possible that respondents (with consent and sufficient information) still had no records in the IAB employment data. Also, even if linkage was possible, it is not guaranteed that administrative data on employment histories were available for the observation period. For example, if people were self-employed or worked as civil servants between 1975 and 2010, or ended their last job as a salaried worker before 1975, data on employment histories were not available. These groups still show up in the administrative records if they once held a job that was subject to social security contributions, but had no data that could be matched to survey data. Because of a rather old sample in our study (55 or older), the proportion of the latter groups may be comparatively high. In sum, this leads to 1927 people with available information on administrative employment histories.

### Measures

Employment histories: an important step is to create harmonized measures on employment sequences in the two sources. This involves state definitions (i.e. employment situations) and equal sequence lengths. Concerning sequence length, sequences between 1975 and 2010 are available in both sources on a yearly basis. With regard to states, both sources allow us to distinguish three employment situations: (1) full-time employed “*E*”, (2) part-time employed “*e*” and (3) not employed “*n*”. The two first states are used for an episode as a salaried employee. For the survey data, full time is assumed if respondents reported that their employment was “full-time (35 h or more)”, and part-time work was assumed otherwise. Following the notification scheme for the administrative data, full-time work is recorded if the contracted hour corresponds to the standard working hours, while part-time work consists of large part-time (18 h or more) and small part-time (less than 18 h) (Antoni et al. 2016). Not employed accounts for any existing gaps between job episodes, including domestic work, unemployment or retirement, but also episodes as a self-employed worker or civil servant. For these aspects, information from both sources is not sufficiently comparable, in particular because periods are only recorded in the administrative data if social benefits are involved (e.g., unemployment benefits).

Additional variables: in addition to sex and age, we include education, two work-related factors (main employment status and main job sector during working life) and two measurements of current health (depressive symptoms and physical inactivity), all taken from survey data.

We distinguish three levels of education according to the International Standard Classification of Educational Degrees (ISCED-97): ‘low’ (pre-primary, primary or lower secondary education), ‘medium’ (secondary or post-secondary education), and ‘high’ (first and second stage of tertiary education). Age (at wave three) is grouped into “55 to 64”, “65 to 74” and “75 years or older”. The two work-related factors refer to the longest job during working life in the survey data (because it is available for the whole sample, irrespective of availability of administrative data). With regard to employment status, “self-employed”, and salaried “employees” are distinguished. The six following categories describe the job sector: “Public service”, “Industry/Mining”, “Craft and trade”, “Sale”, “Other services”, and “Other sectors”. To measure depressive symptoms, we use a binary indicator of increased depressive symptoms, based on the German 15 four-point Likert scaled item version of the Centre for Epidemiological Studies Depression (CES-D) Scale. Increased symptoms are defined as scoring 18 or higher on the sum score (Hautzinger and Bailer 1993), and—in case information is missing in wave 3—we imputed information from prior waves (7% of the cases). Physical inactivity was assessed by a question on whether the respondent reported no involvement in any physical activity within the last 4 weeks.

### Statistical Analysis

We first describe socio-demographic and health-related characteristics of the total sample, and for the parts without and with linked administrative data (all characteristics coming from the survey data). Then, we present trajectory indicators of the employment histories in the two sources (each coming from the respective data source). This includes the average years spent in each state, number of spells and an indicator to describe the general complexity within histories: the “turbulence” (Elzinga and Liefbroer 2007). Higher values refer to more complex histories, and “1” being the least complex history (single state throughout whole observation period). Furthermore, we created “chronograms” for histories from the survey data and from the administrative data, displaying the proportion of each employment situation by year.

We then compare agreements of employment histories and therefore restrict analyses to those with information from both sources (*n* = 1927). At first, we compare the annual level of agreement. Specifically, we compute Cohen’s kappa for each year individually, separately for the three states. This allows us to study if agreements vary across time, and whether agreements are more likely for specific states. Cohen’s kappa is commonly used to test agreement for nominal scales (Cohen 1960). In contrast to simple percent agreement (measuring the proportion of agreement), Cohen’s kappa also accounts for the possibility that an agreement is simply down to chance. Kappa usually ranges between 0 and 1. Values between 0.41 and 0.60 are considered a moderate agreement, 0.61–0.80 as substantial, and 0.81–1.00 as almost perfect agreement. Second, we apply sequence analyses and conduct pairwise comparisons of entire individual employment sequences (Abbott 1995; Aisenbrey and Fasang 2010; Studer and Ritschard 2016). The same approach was adopted in previous studies comparing information on prospectively collected survey data and administrative data (Huber and Schmucker 2009; Manzoni et al. 2010). In contrast to annual Cohen’s kappa, this compares entire sequences from 1975 till 2010 (36 years) and is not restricted to annual comparisons. We calculate two alternative distance measures. The first measure simply counts the number of necessary substitutions to make one sequence equal to the other, often referred to as “naïve distance” or “traditional hamming distance” (with substitution costs = 1). As a result, the calculated distance is equal to the number of years where employment situations are different. As an alternative, we apply optimal matching (OM). This is the most common approach within sequence analyses and has an important feature for our analyses (see Halpin (2012) or Studer and Ritschard (2016) for an overview of distance measures). In contrast to the naïve distance, OM can recognize similarities between two sequences that are only shifted by some years, because it also allows for alignments (“insertion” and “deletion”) when comparing the two sequences (with “indel costs” set to 0.5 in our case^1^). This distance may be more appropriate for our analyses, because some participants may just report the timing of specific episodes incorrectly (but recall the order of their employment histories correctly). In Sect. 3, we show the mean, standard deviation, median and interquartile range by covariates, and a histogram of both measures is presented in Appendix. Finally, we estimate linear regression models (ordinary lease square (OLS) regressions) with each distance as a dependent variable and present estimates of two types of regression models. In model 1, all covariates (socio-demographic, health and work-related factors) are included simultaneously, and model 2 additionally includes the trajectory indicators (based on the administrative histories).^2^ By comparing both models, we can explore the degree to which the association between covariates and distances is due to characteristics of the histories. For ease of interpretation, we use non-transformed distances and present unstandardized estimates. Estimates for transformed distances (square root), though, can be found in Appendix (Supplemental Table 1), and we replicated findings based on Poisson or Tobit regression models (with 0 as lower and 36 as upper limit). Calculations and graphs are based on the SADI package in Stata (Halpin 2014), and the TraMineR package in R is used for calculating distances (Gabadinho et al. 2011; Studer and Ritschard 2016).

## Results

In Table 1, we see that the subsample of people with linked administrative data (right column) is more likely to be male, are slightly younger, and have lower levels of education than the total sample of those with survey data (left column). Those who provide survey data are more likely to be self-employed and tended to work in the public service sector, while people with administrative data rather worked in the primary sector (particularly industry and mining). These latter findings are notably obvious if we contrast people with linked administrative data to those who provided survey data only (middle column). This is to be expected (because those who were continuously self-employed or civil servants are not part of the administrative data). Health-related factors are similar for the different groups, both in terms of depressive symptoms and physical inactivity.

**Table 1:** Comparison of total sample and parts with and without linked administrative data: observations (No.) and frequencies in percentage (Col. %), or mean and standard deviation (SD)

Variables	Total (*n* = 2983)		Without linked adm. data (*n* = 1056)		With linked adm. data (*n* = 1927)	
	No.	Col. %	No.	Col. %	No.	Col. %
Sex						
Male	1472	49.3	443	41.9	1029	53.4
Female	1511	50.7	613	58.1	898	46.6
Total	2983	100.0	1056	100.0	1927	100.0
Age						
55–64 years	1023	34.3	292	27.6	731	37.9
65–74 years	1246	41.8	424	40.2	822	42.7
75 years or older	714	23.9	340	32.2	374	19.4
Total	2983	100.0	1056	100.0	1927	100.0
Education						
Low	1671	56.1	521	49.5	1150	59.7
Medium	586	19.7	190	18.0	396	20.6
High	722	24.2	342	32.5	380	19.7
Total	2979	100.0	1053	100.0	1926	100.0
Employment status^a^						
Self-employed	247	8.4	126	12.3	121	6.4
Employee	2682	91.6	900	87.7	1782	93.6
Total	2929	100.0	1026	100.0	1903	100.0
Job sector^a^						
Industry/mining	676	23.1	146	14.3	530	27.9
Public service	748	25.6	364	35.5	384	20.2
Craft and trade	300	10.2	95	9.3	205	10.8
Sale	560	19.1	194	18.9	366	19.2
Other services	420	14.3	131	12.8	289	15.2
Other sectors	223	7.6	94	9.2	129	6.8
Total	2927	100.0	1024	100.0	1903	100.0
Physical inactivity						
Yes	1154	38.7	416	39.4	738	38.3
No	1829	61.3	640	60.6	1189	61.7
Total	2983	100.0	1056	100.0	1927	100.0
Depressive symptoms						
Yes	198	6.6	51	4.8	147	7.6
No	2785	93.4	1005	95.2	1780	92.4
Total	2983	100.0	1056	100.0	1927	100.0

^a^According to longest job in survey data

Table 2 compares some summary measures of the employment histories for the two sources. We see that the average years spent in full-time employment is comparatively lower in the employment histories from the survey data, particularly for those who provided survey data only (14 years). Furthermore, average years spent in non-employment are higher in the survey data. In addition, the average number of changes between different employment situations (number of spells) and the turbulence are higher in histories from the administrative data than in the survey data. Persons perhaps reduce the complexity of recalled biographies in the self-reported histories case.

**Table 2:** Summary measures of employment histories from survey data and from administrative data

Variables	Survey data (*n* = 2983)		Adm. data (*n* = 1927)	
	Mean	(SD)	Mean	(SD)
Duration (years) in…				
Not employed	14.90	(12.23)	14.19	(9.99)
Part-time employed	3.80	(8.20)	4.04	(6.45)
Full-time employed	17.30	(13.41)	17.77	(11.48)
Number of spells	2.22	(1.18)	3.80	(2.18)
Turbulence	4.01	(2.53)	5.65	(2.42)

**Fig. 1:**
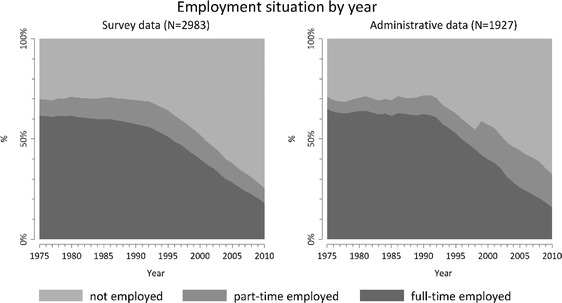
Employment situation by year (chronogram) for survey and administrative data

Figure 1 shows the percentage of each occupational situation for each year of observation for the two sources. The observed patterns are very similar: Rates of full-time employment are above 50% between 1975 and 1995 and decrease thereafter. In the administrative data there is a small sudden increase in part-time employment in 1999. This is due to a change in the notification procedure in 1999, when marginal employments (jobs with very low wages) started being counted as part-time jobs as well (before not counted as job at all).

**Fig. 2:**
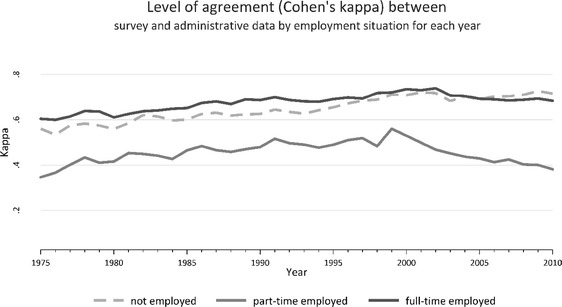
Annual levels of agreement between survey and administrative data by employment situation: Cohen’s kappa

Figure 2 limits the analyses to respondents with data from the two sources (1927 respondents) and investigates levels of agreement in terms of kappa values. Because these are calculated for each year separately, they allow us to study if agreements vary across time. Two findings must be mentioned: First, agreements are only slightly higher in more recent years. Second, levels of agreement are substantial for full-time employment and non-employment (mean kappa of 0.68 in the case of full-time employment and 0.65 for non-employment), but are rather moderate for part-time work (mean kappa 0.45). Perhaps people report more hours within survey data—even if contracted hours are part time.

Table 3 presents the calculated distances between the individual histories (see Sect. 2 for details) according to covariates under study (Table 1). A histogram of both measures can be found in Appendix (Supplemental Fig. 1). Both distances range from 0 to 36, with most people having a score of four or lower. Hence, most people have 4 or less years (out of 36 years) that are different in the two sources. An additional finding is that—although OM distances allow for alignment when comparing the sequences—values for the naïve distances and OM distances turn out to be very similar. This indicates that the timing of episodes matches well in the two sources. When comparing the distances by covariates, we see smaller distances for men, older age groups, those with lower education, and among people without increased depressive symptoms. Furthermore, distances are lower if people were employees in their main occupation or if they worked in the industry or mining sector. These latter findings are additionally investigated in multivariable analyses, where all covariates are included simultaneously to predict distances, and where model 2 explores if these findings are due to trajectory indicators. Results are presented in Table 4.

**Table 3:** Differences between self-reported and administrative employment histories by covariates: mean, standard deviation (SD), median and interquartile range (*n* = 1927)

Variables	Naïve distance				OM distance			
	Mean	(SD)	Median	IQR	Mean	(SD)	Median	IQR
Sex								
Male	5.3	(7.0)	2.0	6.0	4.9	(6.6)	2.0	6.0
Female	9.6	(8.6)	7.0	13.0	8.9	(8.2)	6.0	13.0
Age								
55–64 years	7.7	(8.4)	4.0	9.0	7.1	(8.0)	4.0	9.0
65–74 years	7.5	(8.0)	5.0	10.0	7.0	(7.6)	4.0	10.0
75 years or older	6.2	(7.7)	2.0	9.0	5.7	(7.0)	2.0	9.0
Education								
Low	7.2	(8.0)	4.0	10.0	6.6	(7.4)	3.5	10.0
Medium	7.4	(8.3)	4.0	10.0	7.0	(8.0)	4.0	10.0
High	7.5	(8.3)	5.0	9.5	7.1	(8.0)	4.0	9.5
Employment status								
Self-employed	8.0	(7.9)	5.0	9.0	7.5	(7.7)	5.0	9.0
Employee	7.2	(8.0)	4.0	10.0	6.6	(7.6)	4.0	10.0
Job sector^a^								
Industry/mining	4.7	(5.9)	2.0	5.0	4.3	(5.5)	2.0	5.0
Public service	7.7	(8.9)	4.0	10.0	7.2	(8.5)	4.0	10.0
Craft and trade	7.8	(8.7)	4.0	11.0	7.1	(7.9)	4.0	11.0
Sale	8.2	(7.6)	6.0	11.0	7.5	(7.2)	5.0	11.0
Other services	8.8	(9.0)	6.0	12.0	8.2	(8.6)	5.0	12.0
Other sectors	9.1	(8.4)	7.0	12.0	8.6	(8.1)	6.0	12.0
Physical inactivity								
Yes	7.2	(8.0)	4.0	10.0	6.6	(7.5)	4.0	10.0
No	7.4	(8.2)	4.0	10.0	6.9	(7.7)	4.0	10.0
Depressive symptoms								
Yes	9.1	(8.6)	6.0	12.0	8.3	(8.1)	6.0	12.0
No	7.2	(8.1)	4.0	9.0	6.7	(7.6)	4.0	9.0
Total	7.3	(8.1)	4.0	10.0	6.8	(7.7)	4.0	10.0

^a^According to longest job in survey data

**Table 4:** Results of multivariate analyses predicting naïve and optimal matching (OM) distances: unstandardized regression coefficients (*b*) with levels of significance, standard errors (SE) and confidence intervals (CI 95%) (*n* = 1902)

Variables	Naïve distance						OM distance					
	Model 1			Model 2			Model 1			Model 2		
	*b*	(SE)	CI 95%	*b*	(SE)	CI 95%	*b*	(SE)	CI 95%	*b*	(SE)	CI 95%
Sex												
Male (ref.)	–						–			–		
Female	3.91***	(0.40)	[3.13,4.68]	0.69	(0.44)	[− 0.16,1.55]	3.58***	(0.37)	[2.85,4.31]	0.75	(0.42)	[− 0.07,1.57]
Age												
55–64 years (ref.)	–						–			–		
65–74 years	0.15	(0.40)	[− 0.63,0.93]	− 0.27	(0.38)	[− 1.02,0.47]	0.17	(0.38)	[− 0.56,0.91]	− 0.26	(0.36)	[− 0.97,0.45]
75 years or older	− 0.62	(0.50)	[− 1.60,0.37]	− 1.45**	(0.50)	[− 2.44,− 0.46]	− 0.64	(0.47)	[− 1.57,0.28]	− 1.53**	(0.48)	[− 2.47,− 0.58]
Education												
Low (ref.)	–						–			–		
Medium	− 0.12	(0.46)	[− 1.02,0.78]	0.44	(0.43)	[− 0.41,1.28]	0.09	(0.43)	[− 0.76,0.95]	0.58	(0.41)	[− 0.23,1.38]
High	1.01*	(0.48)	[0.06,1.96]	0.58	(0.46)	[− 0.32,1.47]	1.06*	(0.46)	[0.16,1.96]	0.69	(0.44)	[− 0.17,1.55]
Employment status												
Self-employed	0.05	(0.76)	[− 1.43,1.53]	− 1.66*	(0.73)	[− 3.09,− 0.23]	0.09	(0.71)	[− 1.30,1.50]	− 1.51*	(0.70)	[− 2.88,− 0.14]
Employee (ref.)	–						–					
Job sector^a^												
Industry/mining (ref.)	–			–			–			–		
Public service	1.44**	(0.54)	[0.38,2.49]	0.97	(0.51)	[− 0.03,1.96]	1.41**	(0.51)	[0.41,2.41]	1.02*	(0.48)	[0.07,1.97]
Craft and trade	2.91***	(0.65)	[1.64,4.18]	2.18***	(0.61)	[1.00,3.37]	2.60***	(0.61)	[1.40,3.80]	1.98***	(0.58)	[0.84,3.11]
Sale	1.61**	(0.56)	[0.52,2.71]	0.90	(0.52)	[− 0.13,1.92]	1.47**	(0.53)	[0.43,2.51]	0.85	(0.50)	[− 0.14,1.83]
Other services	2.61***	(0.59)	[1.44,3.78]	1.97***	(0.56)	[0.88,3.06]	2.43***	(0.56)	[1.33,3.54]	1.90***	(0.53)	[0.85,2.94]
Other sectors	2.69***	(0.78)	[1.16,4.22]	2.02 **	(0.73)	[0.59,3.45]	2.69***	(0.74)	[1.24,4.14]	2.08**	(0.70)	[0.71,3.45]
Physical inactivity												
Yes	0.04	(0.37)	[− 0.68,0.76]	− 0.16	(0.34)	[− 0.84,0.51]	− 0.01	(0.35)	[− 0.69,0.67]	− 0.19	(0.33)	[− 0.84,0.45]
No (ref.)	–			–			–			–		
Depressive symptoms												
Yes	0.97	(0.67)	[− 0.35,2.29]	0.68	(0.63)	[− 0.55,1.92]	0.79	(0.64)	[− 0.45,2.04]	0.55	(0.60)	[− 0.63,1.73]
No (ref.)	–			–			–			–		
Duration (years) in…^b^												
Not employed				0.18***	(0.02)	[0.14,0.22]				0.17***	(0.02)	[0.14,0.21]
Part-time employed				0.19***	(0.03)	[0.13,0.25]				0.17***	(0.03)	[0.11,0.22]
Full-time employed (ref.)				–						–		
Number of spells ^b^				0.95***	(0.08)	[0.79,1.11]				0.77***	(0.08)	[0.62,0.92]
Constant	3.70***	(0.50)	[2.73,4.67]	− 0.79	(0.54)	[− 1.85,0.27]	3.40***	(0.47)	[2.48,4.31]	− 0.42	(0.52)	[− 1.43,0.60]
*R*²	0.09			0.21			0.09			0.19		
*N*	1902			1902			1902			1902		

^a^According to longest job in survey data, ^b^based on administrative histories; *p < 0.05, **p < 0.01, ***p < 0.001

In sum, findings are similar to those above, but reveal three important insights: Firstly, once all covariates are considered in model 1, there are no associations for age. Probably, this is due to confounding effects, because particular sectors may be more likely for younger people. Secondly, model 1 again reveals that distances are most pronounced for women and people that mainly work in the craft and trade sector, and for those in other services and sectors not classified (other sectors). The third observation worth noting is that the regression coefficients are generally attenuated, once we include the trajectory indicators in model 2, and that they become nonsignificant in case of women. This result indicates that higher distances for women are largely due to longer time spent in non-employment or part-time employment, and to a higher number of spells—all factors that are related to higher distances. Findings remained unchanged using transformed distances (square root), as presented in Appendix (Supplemental Table 1).

## Discussion

This paper compares two strategies to gain information on previous employment histories in the German HNR cohort study, either retrospectively in the frame of an interview (survey data), or via record linkage of administrative data from the German Institute for Employment Research (IAB). In the analysis, we first compare respondents with self-reported survey data and with administrative data. Then, we investigate the agreement of self-reported survey data and administrative data (covering 36 years of working life), and finally, we test if the agreement depends on socio-demographic, health and work-related factors. In accordance to these three objectives, the main findings are as follows:

We find important sample differences, both in terms of sample sizes and sample compositions. Almost all participants of the cohort study gave retrospective information on employment histories (97%). Administrative data, in contrast, were available for a smaller number of respondents (63%). The lower number of people with administrative data exists because not every respondent gave consent for record linkage and because linkage was not possible for some respondents, for example due to invalid SSN or because data were not available in the administrative data—even if consent was given (e.g., for self-employed). This finding is in line with previous studies (Korbmacher and Schroeder 2013), and it underlines the importance of interviewer training to foster the respondents’ trust in confidentiality when asking for consent. In addition, it shows that linkage of administrative data is complex and includes various stages at which selection can occur (even if consent is given). It must be noted, though, that selections due to missing consent and unsuccessful linkage only occur if administrative data have to be linked with survey data. As such, administrative data may be easier to work with if there is no need to link it with survey data. The high number of people providing survey data shows that retrospective data collections in interviews are a practical way to gather life history data. This corresponds to findings reporting positive experiences of interviewers and that respondents usually like retrospective interviews (Belli et al. 2007; Schröder 2011). Hence, our study suggests that an imperfect linkage of survey data with administrative data on employment histories can lead to smaller samples. At this point, we must also note that self-reported data on employment histories are available for a longer time frame in our study (for each single job of the career, including when self-employed), while information from administrative data is available for employees and from 1975 onwards only (for East Germany since 1991).

Concerning sequence differences between both sources, two main findings exist: The first is that self-reported employment histories are less complex than in the administrative records, with a smaller number of spells and less years spent in full-time employment. This could be due to individual response styles, in particular the tendency to simplify sequences and to underreport periods of unemployment in interviews (Manzoni et al. 2010), but also due to higher levels of precision in the administrative data. The second main finding is that levels of agreement are generally high, and that this is true across the entire period covered (between 1975 and 2010). Possibly, people recall periods of employment with high accuracy, even job episodes that are long ago (at least on a yearly basis). Most people have only 4 or less years (out of 36 years) that are different in the two sources, which correspond to a median level of agreement of 89% of all years.

In the matter of whether levels of agreement differ by specific factors, we found differences for sex (lower for women) and for job sectors, but no such differences for age or health. Compared to industry and mining sectors, differences are higher for people who mainly worked in the tertiary sectors, such as the craft and trade sectors, or the service sector.^3^ These differences by job sectors and sex are weakened in multivariable models (and become nonsignificant in the case of sex), once the number of spells and durations spent in non-employment and part-time employment are included. This indicates that women (and partly those working in the tertiary sectors) probably have more complex employment histories marked by frequent spell changes and longer time spent in non-employment and part-time employment (Widmer and Ritschard 2009), and this explains higher disagreements.

We need, however, to consider some limitations. Firstly, the used administrative data on employment histories rely on the mandatory German notification scheme, and recording procedures do differ between countries and sources of administrative data, each leading to different samples and recorded information. Thus, our results may not necessarily apply to other contexts. Secondly, the emphasis of our analyses is on entire employment sequences and individual comparisons based on sequence analyses. Thereby—to enable comparisons—we must focus on a relatively rough classification and three states of occupational situations (full-time employed, part-time employed and not employed). We could, therefore, not include information on periods of self-employment or on the reason for non-employment (e.g., home or family work). Similarly, we may include additional information when defining the employment situation (e.g., job title or a job classification). In doing so, however, the use of sequence analysis becomes very complex, because the number of possible sequences grows extensively with number of states. In that case, a simple comparison of provided information (irrespective of timing, duration and sequencing) may be more appropriate. Finally, although the interviews are conducted by trained interviewers, and respondents are prepared when retrospective data are collected, a calendar interview may have provided more accurate information (Belli et al. 2007; Berney and Blane 1997).

In conclusion, this study links information on individual employment histories from two different sources: survey and administrative data. We show that both strategies lead to different samples. In case studies which have descriptive purposes, this may be problematic for the generalizability of findings based on administrative data. It may, however, play a minor role when testing associations between aspects of employment histories and health outcome—often an important objective of occupational cohort studies (Batty et al. 2014). In terms of agreements between survey and administrative data, we found high levels, and a clear indication that both strategies provide reliable data on employment histories.

## Electronic supplementary material

Below is the link to the electronic supplementary material.

Supplementary material 1 (PDF 219 kb)

## Footnotes

1 This corresponds to the default setting, where indel costs are half as large as substitution costs. As a test of robustness, we conducted supplementary analyses with different values.

2 Due to multicollinearity we did not include the indicator of turbulence.

3 The predicted percentage of sequence agreement between survey and administrative data for the different job sectors under study is summarized in the Appendix (Supplemental Figure 2).
