# Supplementary material for: Agreement of Self-Reported and Administrative Data on Employment Histories in a German Cohort Study: A Sequence Analysis
Source: Eur J Popul. 2018 Mar 21;35(2):329–46. doi: 10.1007/s10680-018-9476-2 (PMC6497685; doi:10.1007/s10680-018-9476-2)
Supplement: Supplementary file 1 — Supplementary material 1 (PDF 219 kb) [file 10680_2018_9476_MOESM1_ESM.pdf]

Supplementary files

Supplemental Table 1 – Results of multivariate analyses predicting transformed (square root) naïve and optimal matching (OM) distances: unstandardized regression coefficients (b) with levels of significance, standard errors (SE) and confidence intervals (CI 95%)

Variables		*Naïve distance*				*OM distance*			
		b		(SE)	CI 95%	b		(SE)	CI 95%
Sex	Male (ref.)	-				-			
	Female	0.82	***	(0.07)	[0.68,0.96]	0.78	***	(0.07)	[0.64,0.92]
Age	55-64 years (ref.)	-				-			
	65-74 years	0.05		(0.07)	[-0.10,0.19]	0.06		(0.07)	[-0.08,0.19]
	75 years or older	-0.19	*	(0.09)	[-0.37,-0.00]	-0.18	*	(0.09)	[-0.36,-0.00]
Education	Low (ref.)	-				-			
	Medium	-0.02		(0.09)	[-0.19,0.14]	0.01		(0.08)	[-0.15,0.17]
	High	0.23	*	(0.09)	[0.05,0.40]	0.23	**	(0.09)	[0.06,0.40]
Employment status	self-employed	0.14		(0.14)	[-0.14,0.41]	0.13		(0.14)	[-0.13,0.40]
	employee (ref.)	-				-			
Job sector [a]	Industry / Mining (ref.)	-				-			
	Public service	0.14		(0.10)	[-0.06,0.33]	0.14		(0.10)	[-0.05,0.33]
	Craft and trade	0.50	***	(0.12)	[0.26,0.73]	0.47	***	(0.12)	[0.24,0.69]
	Sale	0.33	**	(0.10)	[0.13,0.53]	0.31	**	(0.10)	[0.11,0.50]
	Other services	0.39	***	(0.11)	[0.17,0.60]	0.37	***	(0.11)	[0.16,0.57]
	Other sectors	0.48	***	(0.14)	[0.20,0.76]	0.49	***	(0.14)	[0.21,0.76]
Physical inactivity	Yes	-0.01		(0.07)	[-0.14,0.12]	-0.02		(0.07)	[-0.15,0.11]
	No (ref.)	-				-			
Depressive symptoms	Yes	0.24		(0.12)	[-0.01,0.48]	0.21		(0.12)	[-0.03,0.44]
	No (ref.)	-				-			
Constant		1.56	***	(0.09)	[1.38,1.74]	1.50	***	(0.09)	[1.33,1.68]
R^2		0.11				0.11			
N		1902				1902			

[a] according to longest job in survey data; * $p < 0.05$, ** $p < 0.01$, *** $p < 0.001$

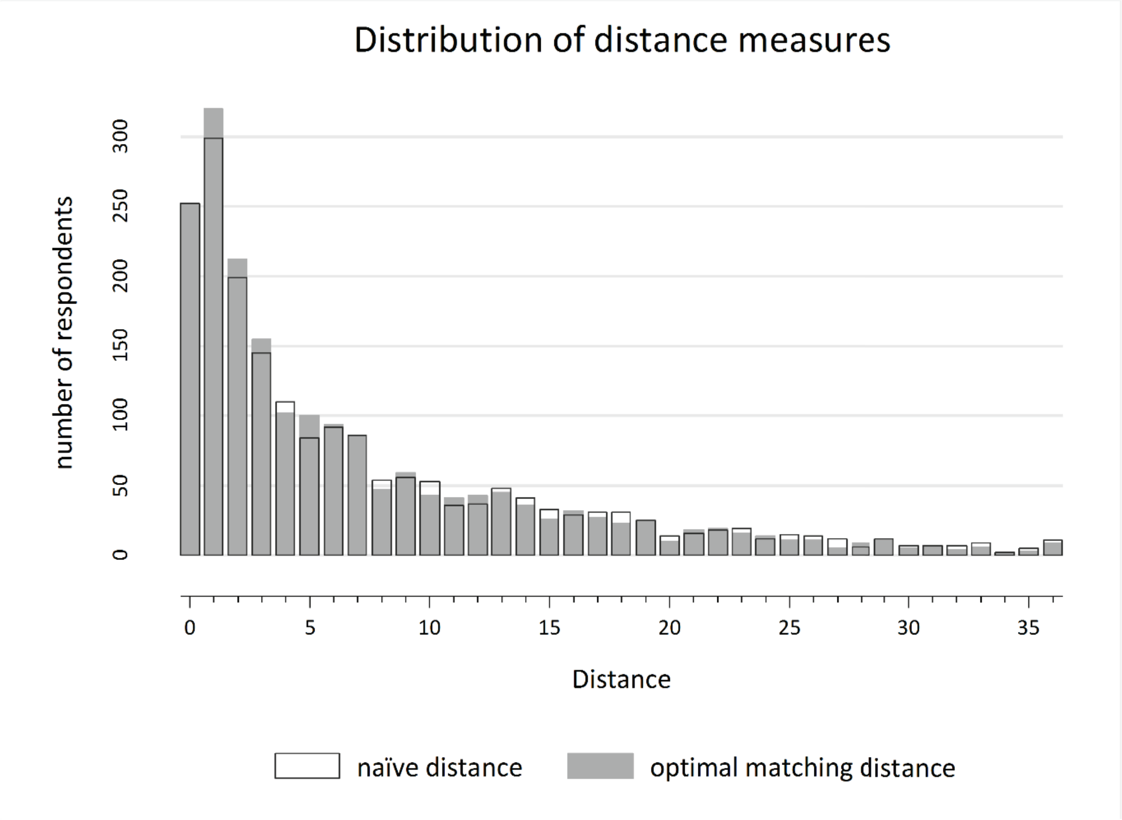

Supplemental Figure 1 – Histogram of distance measures

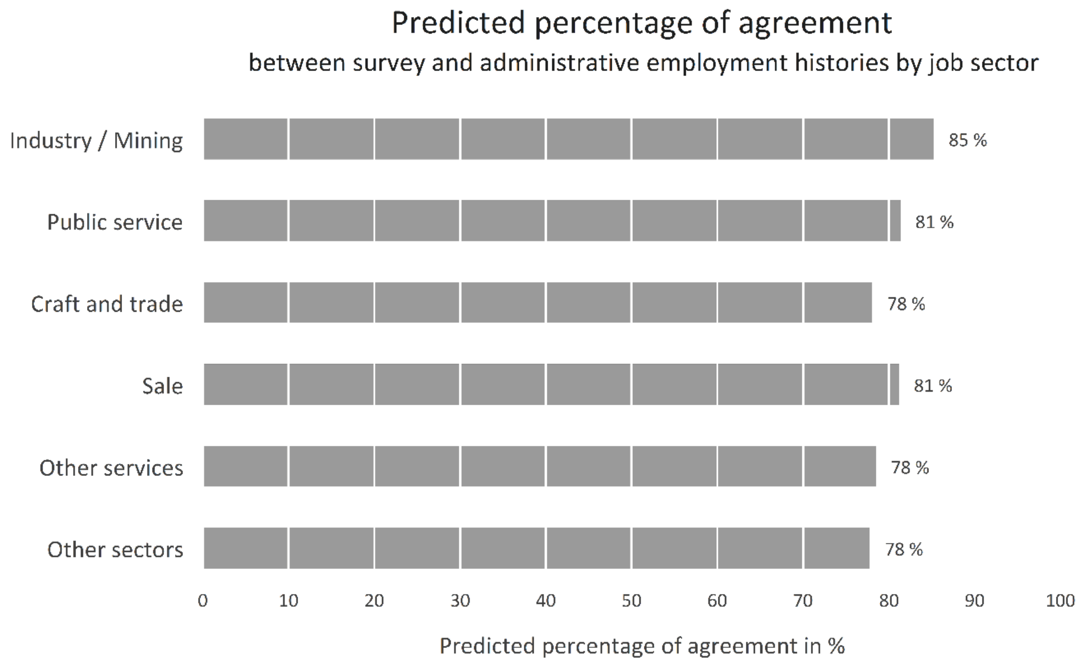

Note. Results are based on results for the OM distance in table 4, in terms of percentage of agreement (100 – (predicted distance / 36 * 100)). The job sector refers to longest held job in survey data.

Supplemental Figure 2 – Percentage of agreement of entire employment histories between survey and administrative data for different job sectors.
